# Correlation Between Serum and Arterial Blood Gas Bicarbonate in Patients Admitted to the Intensive Care Unit

**DOI:** 10.7759/cureus.37703

**Published:** 2023-04-17

**Authors:** Andrew Kim, Leo Yamaguchi, Shobhit Keswani, Vincent Yang, Yi McWhorter

**Affiliations:** 1 Internal Medicine, MountainView Hospital, Las Vegas, USA; 2 Internal Medicine, HCA Healthcare, MountainView Hospital, Las Vegas, USA

**Keywords:** icu, bmp, abg, bicarbonate, acidemia

## Abstract

Objective

Clinicians use two modalities to determine acid-base disturbances: calculated bicarbonate on arterial blood gas (ABG) and measured bicarbonate on basic metabolic panels (BMP). The primary objective was to investigate the discrepancy between the two values for diagnosing acidemia in the intensive care unit (ICU). Our secondary objective was to discern the threshold to treat acidemia within various clinical settings.

Materials and methods

We performed a multi-center study using a retrospective patient chart review consisting of ABG and BMP bicarbonate levels at various pH ranges; 584 adult patients were included in this study. SAS software (SAS Institute Inc., Cary, NC) was used for analysis.

Results

Strong positive correlations were found between calculated ABG and measured BMP bicarbonate, with the group of pH 6.9-7.0 being the strongest. Based on odds ratio analysis, patients were more likely to not receive bicarbonate treatment if pH was greater than 7.1 based on calculated ABG bicarbonate. Patients also did not receive bicarbonate treatment when pH was greater than 7.2 based on BMP bicarbonate levels. Our study found that patients with higher pH (pH > 7.1) were less likely to receive bicarbonate treatment. Patients with pH 6.9-7.0 were more likely to receive bicarbonate treatment. Based on receiver operator curve (ROC) model curves, neither ABG nor BMP bicarbonate values have strong accuracy for diagnosing acidemia.

Conclusion

We found no significant difference in CO_2_ levels and ICU types regardless of if ABG or BMP was used.

## Introduction

When measuring bicarbonate to assess acid-base status in patients, clinicians have two different modalities to guide their management: total serum carbon dioxide (CO_2_) found on a basic metabolic panel (BMP) and calculated arterial blood gas (ABG) bicarbonate. ABG results can be obtained faster when compared to BMP results; therefore, it is an advantageous diagnostic modality for assessing acidemia in the acute care setting.

The total serum CO_2_ is determined by three elements: serum bicarbonate ion (HCO3-), dissolved carbon dioxide (CO2), and carbonic acid (H_2_CO_3_) [1]. The total serum CO_2 _is measured via an enzymatic assay. Because serum bicarbonate ions comprise roughly 95% of the total serum CO_2_ content at physiologic normal states, it serves as a good surrogate for serum bicarbonate [2].^ ^However, if the normal physiology is altered due to stress, disease, or other abnormal conditions, the theoretical ratios between the three elements change. Furthermore, there is no consistent consensus in the literature on how to best quantify these changes [3-5]. In contrast, the calculated ABG bicarbonate values are calculated using Henderson-Hasselbalch’s equation (HCO_3_- = 0.03 x pCO_2_ x 10(pH - 6.1)) as blood gas analyzers do not have the capacity to directly measure bicarbonate from whole blood samples.

In this study, we aim to investigate the discrepancy, if any, between the CO_2 _levels found in BMP and ABG at various physiological states. In addition, we assess the threshold that triggers bicarbonate treatment at specific pH and other clinical settings.

## Materials and methods

Settings and eligibility

This multi-center study used the retrospective chart review of patients’ electronic medical records. The study was approved by the Institutional Review Board (IRB) of Health Corporations of America (HCA) (approval number: 2021-262). After IRB approval and waived informed consent, we included all patients age ≥18 years of age admitted to Health Corporation of America (HCA) hospitals intensive care unit (ICU) between January 1, 2016 and December 31, 2020 who received at least one ABG and one BMP within one hour on the same hospital day. Patients in cardiac (CICU), medical (MICU), neurosurgical (NSICU), and surgical (SICU) ICUs were included in this study. 

Objectives

The primary objective of this study is to investigate the discrepancy between measured serum and calculated ABG bicarbonate values in the ICU. Our secondary objectives are twofold. First, we identify the pH threshold on ABG and BMP that clinicians use to treat acidemia with sodium bicarbonate. Next, we investigate whether or not a specific ICU subgroup receives bicarbonate treatment based on pH levels.

Data collection and outcome measures

The following information was abstracted for each patient: age, gender, ethnicity, International Classification of Disease (ICD)-10 codes, ABG and BMP pH values, base excess, lactic acid, length of stay (LOS), date and time of dialysis if applicable. The ABG auto-analyzers used in this study were the Siemens Rapid Point 500 (Siemens Medical Solutions, Malvern, PA), and the BMP analyzers used were Siemens Dimension Vista 1500 (Siemens Diagnostics, Deerfield, IL). The collection time of ABG and BMP CO2 was within one hour of one another. Data was de-identified and recorded on a password-protected spreadsheet on an encrypted file per institutional protocol.

Sample size calculation and statistical analysis

We performed a retrospective chart review on 3998 patients, and 584 patients’ results were used in the final statistical analysis based on sample size calculation and inclusion criteria listed above. 

Using SAS software, the following parameters were calculated and analyzed. The odds ratio was calculated using the Logistic Regression Procedure as the response variable was binomial (0 = no presence of bicarbonate treatment, 1 = presence of bicarbonate treatment). All available independent variables were accounted for in the regression model. Wald confidence intervals were used to determine significance at the 95% (a=.05) confidence level. Additionally, receiver operator curves (ROCs) were used to determine the diagnostic ability of our model. ANOVA testing was utilized to determine if there was a relationship between NSICU, SICU, MICU, and CICU in bicarbonate measurement and treatment.

## Results

The baseline characteristics of the included 584 patients are provided in Table 1. Of these patients, 212 patients (36.30%) received IV bicarbonate treatment during their hospitalization and 372 (63.70%) did not. Seventy-eight percent of patients were in MICU, and the rest represented the surgical subgroup. PH subsets ranged by 0.1 from 6.8 to 7.3 and above. These subsets were separated for analysis with a left-skewed pattern in which lower pH levels were underrepresented when compared to higher pH levels.

**Table 1:** Patient demographics

Total participants	584
Received IV bicarbonate	212 (36.30%)
Did not receive IV bicarbonate	372 (63.70%)
Ethnicity	
Black	22 (3.77%)
Hispanic	136 (23.39%)
Asian	131 (22.43%)
Caucasian	258 (44.18%)
Other	37 (6.34%)
Gender	
Males	392 (67.12%)
Females	192 (32.88%)
ICU Types	
Cardiac	31 (5.31%)
Medical	459 (78.60%)
Neurosurgical	1 (0.17%)
Surgical	93 (15.92%)
pH	
<6.9	8 (1.37%)
≥6.9 but >7.0	12 (2.05%)
≥7.0 but <7.1	30 (5.14%)
≥7.1 but <7.2	66 (11.30%)
≥7.2 but <7.3	82 (14.04%)
≥7.3	386 (66.10%)
Outcomes	
Expired or hospice	157 (26.88%)
Discharged alive	427 (73.12%)

The patient population in our study was predominantly male (67.12%) and Caucasian (44.18%). Based on the United States Census for 2021, Asian (22.42%) and Hispanic (23.29%) populations were overrepresented while the Caucasian and female (32.88%) populations were underrepresented [6].

**Figure 1:**
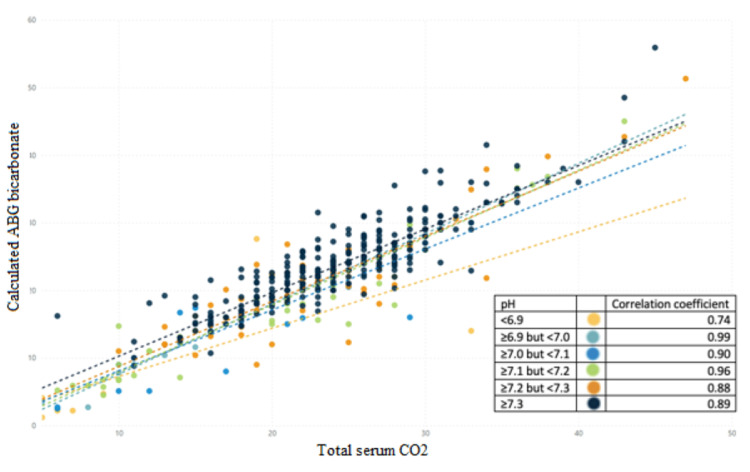
Correlations between CO2 and ABG by pH groups. This graph illustrates the correlation coefficients for various pH groups as outlined in the key located in the lower right of the graph. The closer the coefficient is to 1, the more highly correlated the calculated ABG bicarbonate and total serum CO2 values. CO2 - carbon dioxide, used as a marker for bicarbonate; ABG - arterial blood gas.

There were strong positive correlations between BMP and ABG CO2 levels at all pH ranges. The strongest correlation (0.99) occurred at pH 6.9-7.0, and the weakest correlation (0.74) occurred when pH was less than 6.9 (Figure 1).

**Figure 2:**
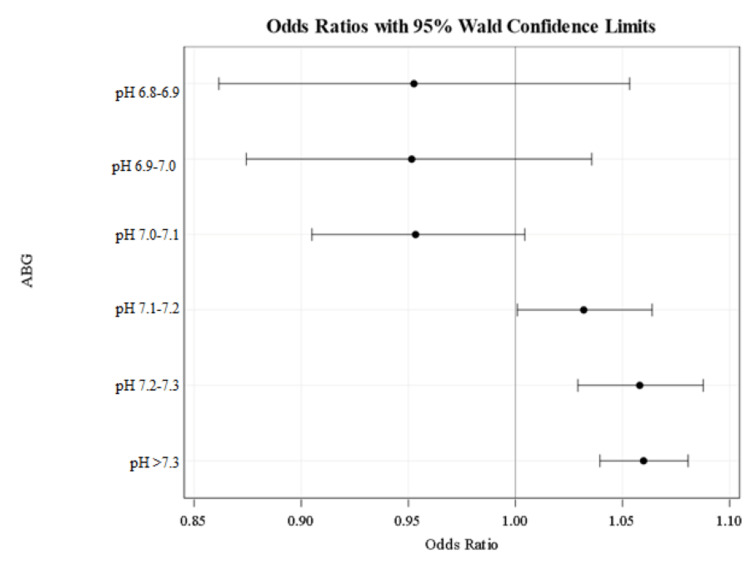
Odds ratios for not receiving bicarbonate treatment when using calculated ABG bicarbonate values. An odds ratio greater than 1 indicates the likelihood of not receiving bicarbonate treatment. An odds ratio of less than 1 indicates the likelihood of receiving bicarbonate treatment. Based on the odd ratios shown above, no pH group was significant in receiving bicarbonate therapy when ABG values were used. ABG - arterial blood gas

**Figure 3:**
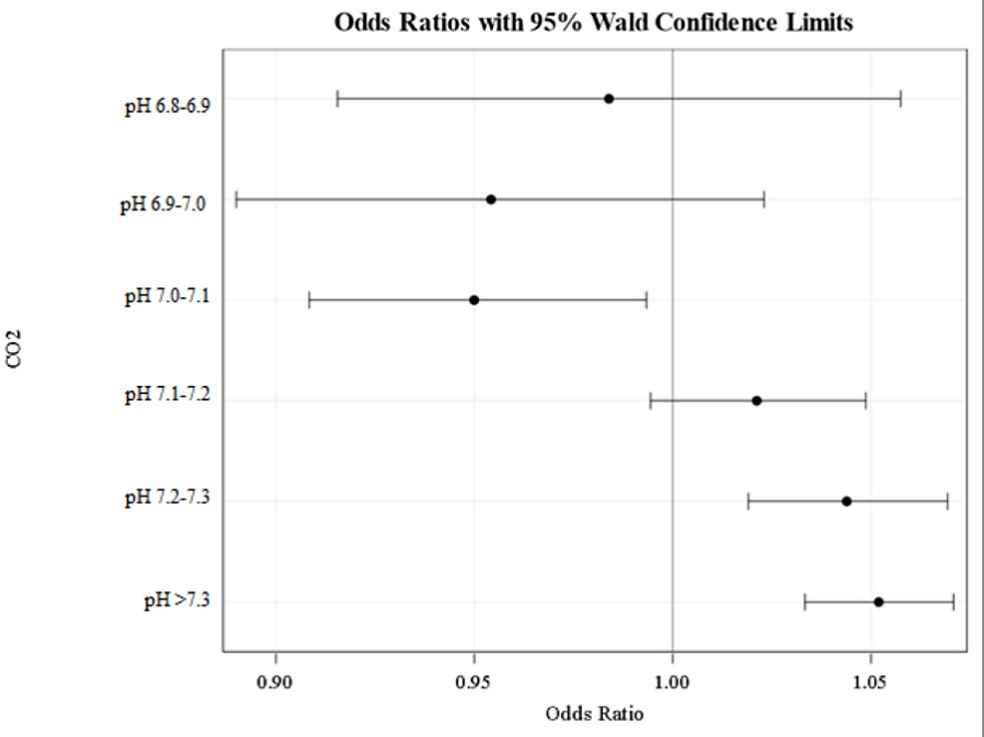
Odds ratios for not receiving bicarbonate treatment when using total serum CO2 values. An odds ratio greater than 1 indicates the likelihood of not receiving bicarbonate treatment. An odds ratio of less than 1 indicates the likelihood of receiving bicarbonate treatment. When using serum CO2, only the pH group of 7.0-7.1 was significant in not receiving bicarbonate therapy. CO2 - carbon dioxide, used as a marker for bicarbonate

The following findings did reach statistical significance with 95% confidence interval. When ABG bicarbonate level was used, if patients’ pH levels were above 7.1, they did not receive bicarbonate treatment. When BMP bicarbonate level was used, patients’ pH level above 7.2 did not receive bicarbonate treatment. Of note, treatment based on serum bicarbonate level in the pH 7.0-7.1 was also significant for patients receiving bicarbonate therapy (Table 2, Figures 2-3).

**Table 2:** Analysis of maximum likelihood estimates for both calculated ABG bicarbonate and total serum CO2. Logistic regressions were used to estimate the odds ratios of not receiving bicarbonate treatment. An odds ratio greater than 1 indicates the likelihood of not receiving bicarbonate treatment. An odds ratio of less than 1 indicates the likelihood of receiving bicarbonate treatment. ABG - arterial blood gas; CO2 - carbon dioxide.

Analysis of Maximum Likelihood Estimates						
Parameter	pH range	Estimate	Standard error	Wald chi-square	P-value	Expected
Calculated ABG bicarbonate	≥6.8 but <6.9	-0.0485	0.0513	0.8958	0.3439	0.953
	≥6.9 but <7.0	-0.0497	0.0432	1.3226	0.2501	0.952
	≥7.0 but <7.1	-0.0477	0.0266	3.2256	0.0725	0.953
	≥7.1 but <7.2	0.0314	0.0155	4.0855	0.0433	1.032
	≥7.2 but <7.3	0.0564	0.0141	16.0105	<0.0001	1.058
Total serum CO_2_	≥6.8 but <6.9	-0.0162	0.0368	0.1946	0.6591	0.984
	≥6.9 but <7.0	-0.0469	0.0355	1.7421	0.1869	0.954
	≥7.0 but <7.1	-0.0514	0.0228	5.0700	0.0243	0.950
	≥7.1 but <7.2	0.0209	0.0135	2.3903	0.1221	1.021
	≥7.2 but <7.3	0.0429	0.0123	12.2381	0.0005	1.044

ROC curves were used to determine the diagnostic efficacy of calculated ABG and measured BMP bicarbonate values. Based on the area under the curve (AUC) of these models, both ABG (AUC = 0.68) and BMP (AUC = 0.67) bicarbonate values have low diagnostic accuracy for acidemia.

**Figure 4:**
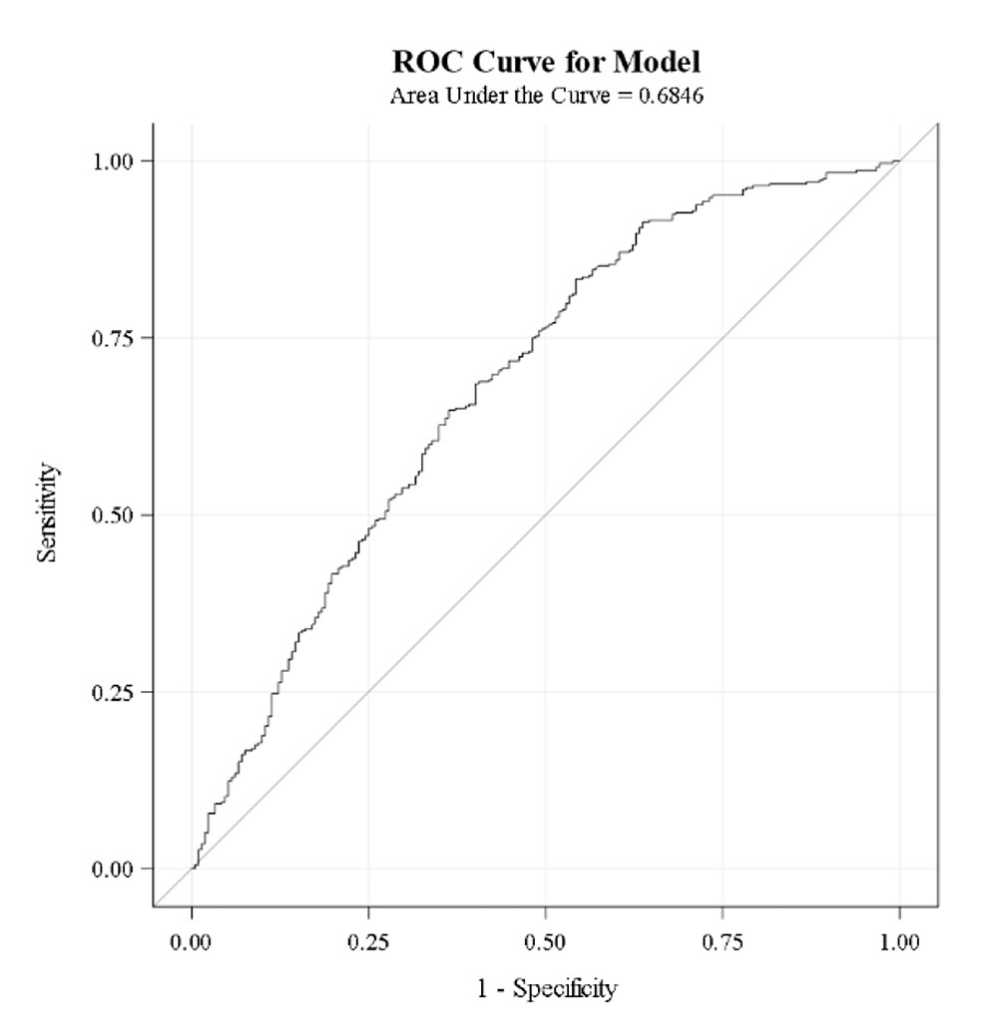
ROC curve for using calculated ABG bicarbonate values to diagnose acidemia. AUC = 0.6846 which suggests that calculated ABG values have low diagnostic accuracy. ROC - receiver operating characteristic; ABG - arterial blood gas; AUC - area under curve.

**Figure 5:**
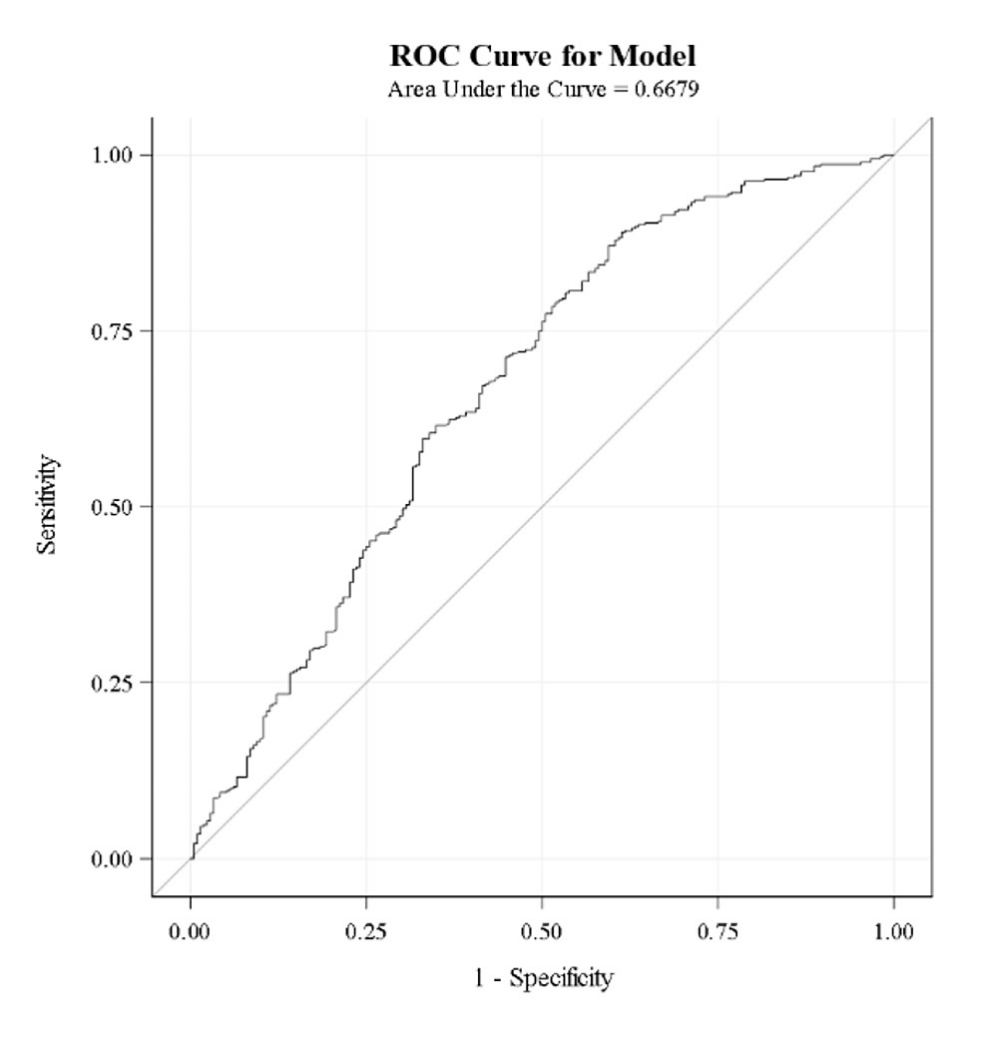
ROC curve for using measured BMP bicarbonate values to diagnose acidemia. AUC = 0.6679 which suggests that total serum CO2 values have low diagnostic accuracy. ROC - receiver operating characteristic; BMP - basic metabolic panel; AUC - area under curve.

There was no statistically significant association between ICU types (MICU, SICU, NSICU, CICU) and bicarbonate values based on calculated ABG or measured BMP (Table 3, Figures 4-5). 

**Table 3:** ANOVA tests for determining whether or not there is an association between ICU types and CO2 levels using ABG and BMP. ABG - arterial blood gas; BMP - basic metabolic panel; ANOVA - analysis of variance.

Source	DF	ANOVA SS	Mean Square	F-value	p-value
Calculated ABG	3	132.4708120	44.1569373	0.88	0.4490
Measured BMP	3	325.4589637	108.4863212	2.60	0.0511

## Discussion

We found a very strong positive correlation between total serum CO2 and the calculated ABG bicarbonate values at all ranges of pH except when the pH is < 6.9. It has been postulated that the dissociation constant of blood carbonic acid (pK’) may vary in the acutely ill and account for the discrepancy seen in our study [7]; however, the difference in pK’ at varying pHs is not large enough to explain large differences between the total serum CO_2_ and calculated ABG bicarbonate even in critically ill patients [8-11]. After a thorough literature review, there is no consensus on why this relationship may exist. It is our hypothesis that physiologic ratios between serum bicarbonate ion (HCO3-), dissolved carbon dioxide (CO_2_), and carbonic acid (H_2_CO_3_) are disturbed at very low pH levels. This could be due to an increased amount of dissolved carbon dioxide and/or carbonic acid levels. Further studies need to be elucidated to understand why calculated ABG bicarbonate is not an ideal surrogate for the total serum CO_2_ at extreme acidic pH.

Sodium bicarbonate treatments for acidemia are common practice among clinicians to quickly correct metabolic and hemodynamic instability, especially in the ICU setting. In our study, we found a general trend that patients with higher pH (> 7.1 in the ABG group and >7.2 in the BMP group) were not likely to receive bicarbonate therapy. These values were found to be the treatment threshold in our study. Only one subgroup was statistically significant for receiving bicarbonate therapy (BMP group pH 7.0-7.1) for acidemia.

We would expect patients with extreme acidemia (pH 6.9-7.0 and pH <6.9) to need bicarbonate therapy. However, analyzing this range did not show any statistical significance. This may be due to a lack of power as there were only 8 patients in this group. We would expect the odds ratio to shift towards a tendency to give bicarbonate treatment when pH < 6.9 if more patients in this pH subset were analyzed.

Based on the low AUC seen on our ROC curve models, neither ABG bicarbonate nor serum bicarbonate had high diagnostic accuracy for treating acidemia. This finding may be due to small sample sizes at lower pH subsets. The AUC and diagnostic accuracy may improve and/or differentiate as sample sizes at lower pH increase.

Our analysis revealed that there was no statistically significant association between ICU type and CO_2_ values when BMP and ABG modalities are utilized. However, there was a trend of differentiation when BMP CO_2_ levels were used to compare different ICU types (p=0.05). The distribution of our ICU subpopulations was heterogenous and likely impacted the results of this outcome: 31 in CICU, 459 in MICU, one in NSICU, and 93 in SICU. Data collection using ICD-10 codes could have contributed to the uneven distribution among different types of ICUs.

Limitations

Because of the retrospective nature of our study, only association rather than causation can be determined. In addition, retrospective studies are prone to confounding, selection, and misclassification biases. Confounding variables such as no randomization and differences in severity of disease were addressed with a wide inclusion criteria. Temporal relations between variables are also difficult to assess. For example, it is difficult to determine the sequence of events in bicarbonate administration and laboratory draws even though we limit a single event to a one-hour duration.

Another important limitation of our study was highlighted by small sample sizes for pH at or below 7.1 and in the SICU subgroup. Therefore, results drawn from acidemic patients need to be interpreted with caution, and future studies need to be conducted with larger sample sizes for patients with pH < 7.2.

## Conclusions

In conclusion, we found that clinicians did not treat patients with sodium bicarbonate if pH > 7.1 on ABG or pH > 7.2 on BMP; however, patients were more likely to receive treatment if serum bicarbonate was between pH 7.0-7.1. Overall, there was no difference in treatment when acidemia was measured with ABG or serum bicarbonate. Future studies with larger sample sizes can offer further insights for treating acidemic patients with pH < 7.2.
